# An Opportunistic Assessment of the Impact of Squirrelpox Disease Outbreaks upon a Red Squirrel Population Sympatric with Grey Squirrels in Wales

**DOI:** 10.3390/ani12010099

**Published:** 2022-01-01

**Authors:** Craig M. Shuttleworth, David Everest, Paul Holmes, Suzi Bell, Rachel Cripps

**Affiliations:** 1 School of Natural Sciences, Bangor University, Bangor LL57 2UW, UK; 2 APHA Weybridge, Addlestone KT15 3NB, UK; david.everest@apha.gov.uk; 3 APHA Shrewsbury, Veterinary Investigation Centre, Shrewsbury SY1 4HD, UK; paul.holmes@apha.gov.uk (P.H.); suzanna.bell@apha.gov.uk (S.B.); 4 Lancashire Wildlife Trust, Bamber Bridge PR5 6BY, UK; rcripps@lancswt.org.uk

**Keywords:** squirrelpox virus, red squirrel, grey squirrel, pathogenic infection, invasive species, disease outbreak

## Abstract

**Simple Summary:**

In Europe, squirrelpox virus is carried by non-native grey squirrels and spread into native red squirrel populations. The virus causes a large proportion of infected red squirrels to die and contributes to local declines and the replacement by grey squirrels. There are relatively few published studies quantifying the impact of disease amongst red squirrels. We present findings from a short-term study in north Wales, United Kingdom.

**Abstract:**

Native red squirrels (*Sciurus vulgaris*) persisted in the coastal mainland woodlands of northern Gwynedd whilst sympatric with an invasive grey squirrel (*Sciurus carolinensis*) population suppressed by culling. Squirrelpox disease in the red squirrel population was recorded in 2017 and 2020/21. An autumn 2020 outbreak was associated with only 17.4% of animals caught and marked in the preceding June known to be present in March 2021. Despite an opportunistic data collection lacking the rigour of empirical experimental design, we observed low local survival rates similar to previously published accounts reported during major squirrelpox outbreaks. The use of a conservation dog to detect red squirrel carcasses resulted in positive detection and confirmation of a temporal and spatial expansion of one disease outbreak. The study is the first in Wales to use conservation dogs and the findings reinforce the vital strategic importance of geographical isolation reducing sympatry of red with grey squirrels in European regions where the introduced congener is a source of the squirrelpox infection.

## 1. Introduction

The introduced Eastern grey squirrel (*Sciurus carolinensis*) causes decline in native Eurasian red squirrel (*Sciurus vulgaris*) populations through resource competition [1]. In Italy there is also spillover of a macroparasite (*Strongyloides robustus*) from the introduced to native sciurid, and infected red squirrels have lower survival rates than those that are not infected [2]. Squirrelpox virus (SQPV) is absent from mainland Continental Europe [1].

SQPV is essentially an asymptomatic infection in grey squirrels [3,4]. Infected populations typically show high viral seroprevalence [5] with pathogenic cases extremely rare [6]. The virus occurs within the native North American population and is also found within some introduced populations in Britain and Ireland [5,7,8,9]. In these areas of Europe, inter-specific infection into sympatric native red squirrel populations produces disease [10,11,12,13]. Infected individuals develop viral-laden lesions, particularly around the lips and eyes and on the chin and muzzle [14], areas where secondary infection by *Staphylococcus* spp. is often present.

Histological examination of a skin lesion was described by McInnes et al. [12] as an exudative dermatitis with ballooning degeneration in the stratum spinosum at the periphery of severe necro-suppurative dermatitis resulting in full-thickness ulceration of the epidermis. Inter-specific viral infection from grey to red squirrel often results in extensive red to red squirrel intra-specific spread because levels of viral shedding from such lesions are high [15]. Viral shedding poses a threat via direct contact between conspecifics and indirect infection via exposure to environmental contamination [12,16].

The highly pathogenic infection may lead to a large proportion of affected red squirrels dying [17,18]. Outbreaks therefore accelerate the rate of replacement of red by grey squirrels in a landscape [19,20] alongside the parallel negative effect of direct resource competition [21]. Consequently, squirrelpox presents a major challenge to red squirrel conservation in Britain and Ireland [22]. Typically, disease outbreaks occur repeatedly in red squirrel populations sympatric with grey squirrels, although the scale and temporal pattern of outbreaks will vary [15,23]. It is also notable that although SQPV antibodies were detected in some regional grey squirrel populations, disease was not observed in sympatric red squirrels for many years [7]. It is recognised however, that this finding could reflect the logistical challenges in detecting every outbreak in extensive landscape areas [12].

**Figure 1:**
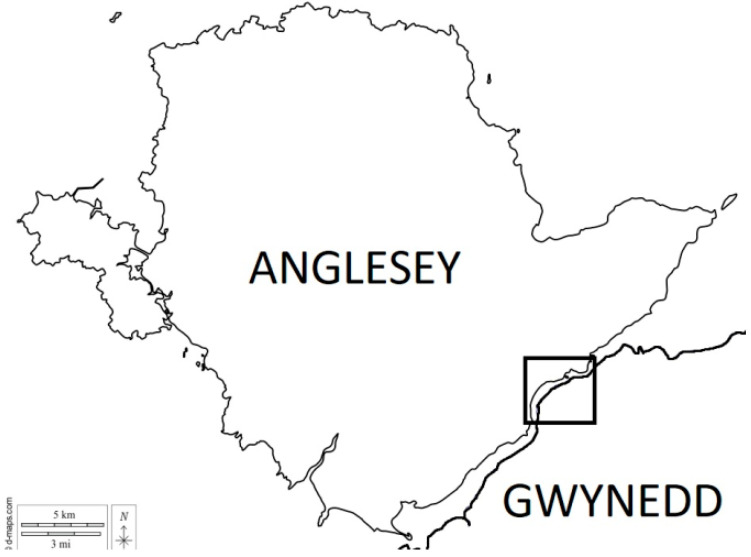
The island of Anglesey and north Wales coast separated by the Menai Strait sea channel. The mainland study area is demarked by a black box.

Reducing the inter-specific infection risk requires the early detection and removal of grey squirrels present in red squirrel areas [24,25]. In addition, the detection of disease in red squirrels is reliant upon a constant vigilance, with visual surveillance and population monitoring [26]. Whilst our ecological understanding of viral epidemiology is sufficient for effective proactive monitoring and reactive contingency planning, in practice such monitoring and associated intervention in the field can face logistical barriers. A variety of factors can affect the efficiency of disease management in red squirrel populations not least the extent to which land is accessible, the level of resources available and the relative landscape-scale grey squirrel dispersal pressure. To illustrate these realities, we report on the challenges experienced when dealing with squirrelpox outbreaks within northern coastal mature mixed woodlands in the administrative county of Gwynedd, north Wales (Figure 1).

The current coastal Gwynedd red squirrel population is sympatric with grey squirrels that first established in the 1960s. Red squirrels had in fact historically become extinct in this area, but then naturally re-colonised following dispersal from the adjacent island of Anglesey. The first red squirrels were recorded on the mainland coast in 2009 having crossed the narrow Menai Strait sea channel. Serological Enzyme-Linked Immunosorbent Assay (ELISA) screening has revealed that 69% of healthy grey squirrels tested were positive for SQPV antibody presence [27].

Anglesey contains the largest red squirrel population in Wales following the successful eradication of grey squirrels [28]. Island red squirrel recovery followed grey squirrel removal and was accelerated through the translocation of additional animals to augment a small residual population [29,30]. The grey squirrel eradication was to a narrow coastal sea-channel boundary with subsequent reinvasion an ongoing and high-risk possibility [28]. This threat is reflected by the fact that a total of eight grey squirrels were detected and removed from Anglesey in the period 2015 to 2021.

Grey squirrels found on Anglesey were legally trapped and culled to prevent the spread of squirrelpox infection into the sympatric red squirrel population. In contrast, the risk of intra-specific infection occurring between the mainland and island red squirrel populations is harder to manage because individual movement across the Menai Strait cannot realistically be prevented and, as animals are fully protected by law, they cannot be trapped without a license. The risk of an infected mainland red squirrel crossing the sea channel and bringing infection onto Anglesey from Gwynedd is ever present and additional to the risk of infection being spread via grey squirrel dispersal.

Regional red squirrel conservation requires the rapid detection and removal of grey squirrels from Anglesey, the suppression of mainland grey squirrel populations and the monitoring of red squirrels for signs of disease [24]. On the island, public awareness of reporting requirements was high. Visual monitoring of red squirrels visiting supplemental feeding points gave confidence that squirrelpox outbreaks were not occurring, whilst reports of grey squirrel sightings were paramount in reinvasion detection and subsequent capture and removal of the animals [25]. The mainland situation was much more complex as here few people reported grey squirrels because the species was so commonplace. Mainland red squirrel monitoring was heavily reliant upon opportunistic captures made during grey squirrel control operations as there was no garden feeding of red squirrels. Red squirrels caught during live trapping operations were measured and weighed to assess individual body condition. The public were encouraged to report any casual sightings animals via social media, email or telephone, and especially evidence of sick or dead red squirrels [13,26]. Volunteers had trail-cameras monitoring five fixed forest locations and reported red squirrel detections that suggested disease.

## 2. Materials and Methods

### 2.1. Land Access

**Figure 2:**
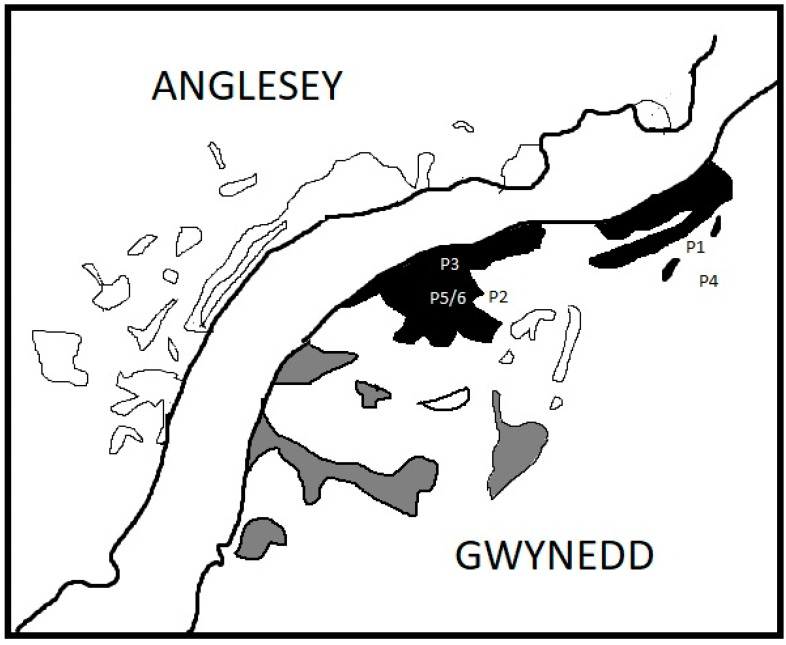
Locations of woodlands, in the mainland study area, not trapped (outlined); trapped with all year access (black shade) and with restricted access (grey shade). Confirmed squirrelpox case locations are shown P1 (7 September 2017), P2 (27 September 2017), P3 (22 November 2017), P4 (29 September 2020), P5 (18 October 2020) 7 P6 (31 January 2021).

Annual trapping could only be conducted in a proportion of the mainland woodland habitat between March and July because of game-rearing operations taking place (Figure 2). The restrictions reduced trapping from 27 to 15 trap locations. In addition, public access to the area was limited to the use of footpaths and hence volunteers were unable to undertake any conservation activity.

### 2.2. Post-Mortem and Laboratory Tests

Red squirrel carcasses showing signs suggesting a pathological skin infection were submitted to the Animal and Plant Health Agency (APHA), Shrewsbury Veterinary Investigation Centre for gross post-mortem and associated laboratory testing. Examinations were undertaken within the APHA Diseases of Wildlife Scheme, a part of the GB Wildlife Disease Surveillance Partnership.

Where appropriate, a sample of skin lesion was then submitted to the APHA Weybridge laboratory for Transmission Electron Microscopy (TEM) screening as described elsewhere [31], amplification of viral deoxyribonucleic acid (DNA) using Polymerase Chain Reaction (PCR) (see [32]) and ELISA tests (see [5]).

The location of each recovered carcass was recorded. The cumulative data allowed the construction of the timeline of squirrelpox infection confirmation in red squirrels. All animals had a submission reference code which provides a unique catalogue entry for all pathological examinations and histology.

### 2.3. Carcass Recovery

Resource limitations precluded regular and systematic proactive ground searches to be undertaken to detect and recover dead squirrels. Instead, the regional conservation programme relied upon initial opportunistic discovery of carcasses by members of the public or contractors undertaking grey squirrel control. Where subsequent laboratory tests revealed squirrelpox infection, then limited searches were undertaken focusing on areas known to have high red squirrel activity. In one instance, a search was undertaken using a conservation dog trained to find dead red squirrels [33]. This single-day monitoring resource was generously provided without an associated financial cost.

### 2.4. Trail Cameras

Volunteers managed trail-cameras that monitored animal activity at point locations baited with sunflower seed. This encompassed five discrete and permanent woodland locations selected because (1) landowner permission allowed round the year access and (2) because the locations were away from areas with high public recreational activity (mountain biking, dog walking or walkers). Volunteers would share recorded images suggestive of squirrelpox infection in a red squirrel. Camera trap models varied and both video and still image settings were used interchangeably.

### 2.5. Live Trapping

Live trapping involving the handling of captured red squirrels could only be undertaken by a licensed professional ecologist. This individual had limited time resource. Unmodified commercially available Albi™ single-catch Mink-traps baited with hazel nuts and sunflower seed were deployed. Traps were cleaned with Virkon S™ disinfectant between all captures. All traps were opened in the morning and closed after inspection three to four hours later. From June 2020 to September 2021, captured red squirrels were handled by transferring them into a plastic bag and then into a Virkon S™ sterilised wire mesh handling cone. Individuals were sexed, weighed to the nearest 5 g with a 1000 g Pesola™ spring-balance, had their hind shin measured to the nearest 0.1 mm and reproductive state categorised in earlier studies [34,35]. These were non-breeding (vulva small, no longitudinal opening), oestrus (vulva swollen and reddish in colour), pregnant (vulva is initially still enlarged, with the longitudinal opening progressively closing to leave a clearly defined central line), lactating (nipples enlarged with milk excretion on stimulation) and post lactation (nipples darkened, hair loss in surrounding area significant often with regrowth).

Measurements collected helped to gauge body condition using weight/skeletal size ratio. However, in the light of subsequent squirrelpox outbreaks, the information provided a useful insight into the relative conditions of groups of detected and undetected animals. Given that squirrelpox is likely spread by direct and indirect (environmental surface contamination) we predicted that because larger sized animals may be more dominant they are therefore most likely to encounter infection.

A unique Passive Integrated Transponder (PiT tag) (Peddymark™ 1.4 × 10 mm) was inserted under the skin at the nape of the neck or dorsal area of the back where the skin was relatively loose. This facilitated individual identification.

Captured red squirrels were examined for signs of external disease. Red squirrels showing signs of infection were taken to a local veterinary practice for examination and professional assessment. When squirrelpox was confirmed in the population, handling of captured red squirrels was limited to reduce the risk of cross infection. Each squirrel was able to move into a bag placed to cover and enclose the front of the trap and the trap door, and when inside the bag, it was scanned for a PiT tag using a Peddymark™ ISO PM450 Scanner. Chipped animals were released unless earlier casual observation of the squirrel in the trap had suggested a potential lesion presence. Unmarked animals and those marked individuals that were to be more closely examined for signs of infection were placed in the handling cone and measured. The approach sacrificed collection of biometric data in order to reduce exposing animals to stress and limiting opportunities for accidental viral cross infection.

Trapping interventions aimed to reduce grey squirrel abundance but, were not sufficient to eradicate grey squirrels. Any captured grey squirrel was euthanized using the cranial dispatch method [36]. Grey squirrels were measured, sexed and had their reproductive state categorised using the same methods as described by [34,35] for red squirrel.

Adult red squirrels were defined as individuals with a weight greater than 240 g. Adult grey squirrels were 450 g or greater in weight.

Finally, data from a third-party seasonal contractor undertaking grey squirrel control were also available. The contractor was not licensed to handle captured red squirrels, but records of incidental trapping were recorded which provided a measure of return per unit trap effort.

## 3. Results

**Figure 3:**
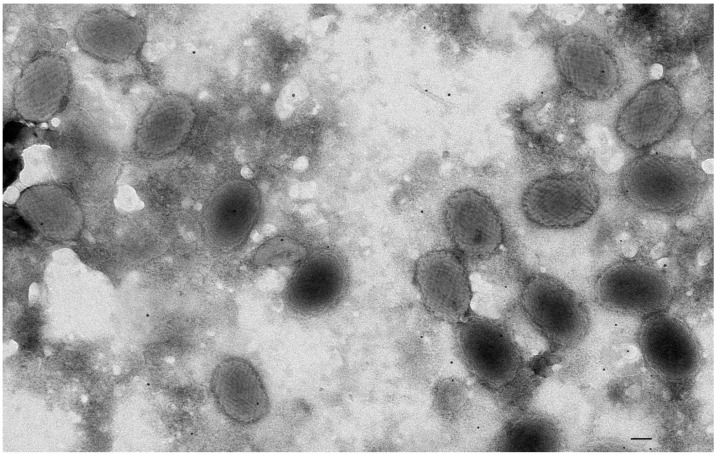
Negative contrast stain transmission electron micrograph displaying SQPV particles in facial skin lesion material from the first confirmed red squirrel squirrelpox case in Gwynedd from Treborth. Bar = 100 nm.

On 7 September 2017, a dead male (P1) red squirrel (APHA Ref 26-M0286-09-17) weighing 282 g was discovered in a live-capture trap being operated in Treborth Botanical gardens as part of ongoing grey squirrel control (UK grid reference SH 5505 7097). There was a small minor lesion on the lip. Hair loss was observed around the right eye, right whiskers and chin surrounding the bottom lip and at the margin between the foot pads and hairy skin on both front feet. The skin surrounding the right eye was noticeably pale and thickened, covering a circular area of approximately 1 cm diameter. Histological examination documented typical poxviral inclusions in keratinocytes within hair follicles, supporting a diagnosis of SQPV. TEM (Figure 3) revealed numerous particles that were of a size, shape and had visible surface morphology consistent with that of SQPV in the lesion. This was the first confirmed squirrelpox case in Gwynedd. An ELISA serological test for SQPV antibodies was negative although the value 0.1995 was only just below the 0.2 positive-test threshold.

Three weeks later, 27 September 2017, the carcass of a female (P2) (APHA Ref 26-M0280-10-17) weighing 244 g was recovered. A member of the public discovered the remains on the woodland floor within the Faenol estate (SH 539 702). Gross post-mortem recorded reddening of the skin present on the head in well circumscribed areas on the lower chin and lip extending to the left and right sides and a circular area on the right side of the face below the right nostril, approximately 5 mm diameter and the right upper lip. Affected areas were reddened and thickened with a crusty surface and some hair loss. There was also reddening around both eyes and of the skin of the digits of both front feet. Histology observed marked, diffuse orthokeratotic hyperkeratosis and scaling. The epidermal surface was colonised by large numbers of cocci, which were also found multifocally within follicles and the dermis, but without any inflammation (post-mortem growth). Haemolytic coagulase positive *Staphylococcus aureus* and non-haemolytic *Escherichia coli* were found to be present. SQPV particles were observed within lesions via TEM.

On 22 November 2017 the remains of a 410 g female (P3) red squirrel (APHA Ref 26-M0440-11-17) were found following a search near a camera location (UK grid reference SH5351 7048) from which images of a red squirrel with swollen eyes were recorded. Gross post-mortem noted the lower lip was swollen and there was an area of ulceration with a flap of necrotic skin extending from the bottom of the lower lip under the jaw approximately 1 cm diameter. Lesion material taken from around the eye and from the lip were both confirmed TEM positive for SQPV particles.

A grey squirrel control contractor trapped in the Faenol woodland in February/March 2017, June/July 2017 and January/February 2018 and reported 1.79, 4.73 and 5.92 grey squirrel captures per 100 days trap effort and 1.14, 0.81 and 2.96 red squirrel captures per 100 days trap effort. He reported red squirrel captures throughout the area denoted by black in Figure 2 in the late winter/spring 2018. There was no evidence of red squirrel extinction or a decline in population distribution, however the overall impact of disease could not be quantified.

**Figure 4:**
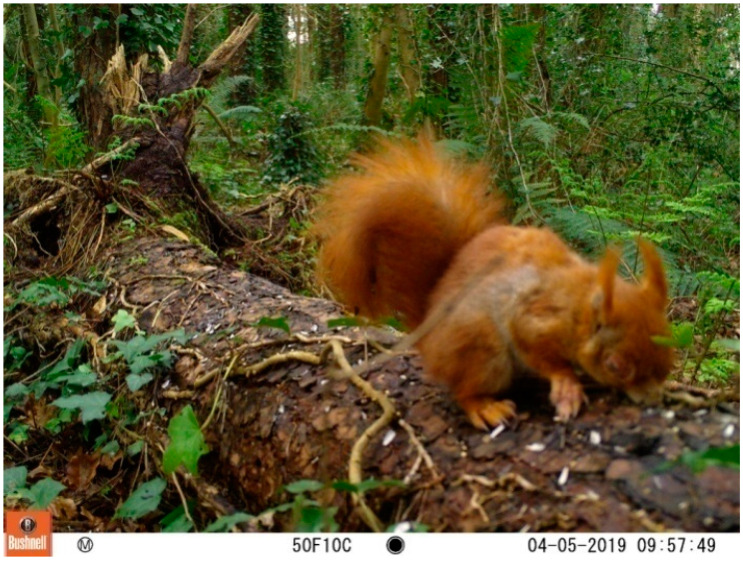
2019 Trail-camera image (5 April 2019) of a red squirrel displaying significant pathogenic skin disease. No body was recovered, and hence pathological investigation was precluded.

On 5 April 2019 a member of the public sent in images from a wildlife camera within Treborth gardens which showed a red squirrel with lesions around the eyes and on the digits (Figure 4). A visual search of the area by volunteers failed to recover a body and therefore it was not possible to determine the cause of the observed condition.

In the summer of 2020, live trapping across Faenol and Treborth (Figure 2) over eight days (in the period 11–18 June) using 27 traps (representing 216 days trap effort) removed 24 grey squirrels. Six of the seven adult females (555.7 g ± 12.2 s.e., *n* = 7) were lactating and were caught alongside a subadult female. Of 13 adult males (524.2 g ± 11.8 s.e, *n* = 13), 10 had large scrotal testes and the mature animals were removed along with three subadults (<450 g). A total of 19 female and 15 male sympatric red squirrels were caught and marked (Figure 4). This included four pregnant and three lactating females, and eight adult males with scrotal testes. A single young male was caught. There were 29 red squirrel recaptures of marked animals. Adult males weighed 308.6 g ± 11.8 s.e. (*n* = 14) whilst mean female weight (excluding four pregnant animals) was 308.6 g ± 10.0 s.e. (*n* = 15).

Later that year, on 29 September 2020, a member of the public found a dead adult female (P4) red squirrel (APHA Ref 26-M0037-10-20) weighing 240 g within the Eithinog Nature Reserve (adjacent Treborth gardens). The carcass showed skin lesions characteristic of squirrelpox infection which was confirmed via TEM. Given the difficulties in searching for red squirrel bodies in woodland experienced in 2019, it was decided to use a conservation dog trained to recover dead red squirrels. On 18 October 2020 a systematic search was undertaken by ‘Max’ and his handler, both fully trained by Kryus Ltd (Formby, UK). Red squirrel remains (P5) in the form of part of tail and clumps of body hair were found in a localised area within Faenol estate. PCR testing confirmed the presence of amplified SQPV DNA. This was the second confirmed outbreak in Gwynedd. Unfortunately, in the autumn of 2020 two cameras were stolen and because of COVID-19 restrictions, volunteers were unable to monitor the remaining cameras and they were removed to prevent further theft.

Temporal land-access restrictions meant that significant areas were also inaccessible and therefore only a proportion of the woodland area trapped in June could be surveyed using traps from the autumn (Figure 2). Within the accessible woodland fragments, the earlier June 2020 trapping (15 traps in eight days equivalent to 120 trap days) caught five grey squirrels (0.04 per trap day) and 23 individual red squirrels (0.19 per trap day excluding recaptures).

In October (30 September 2020–21 October 2020) the same survey effort (120 trap days across the same 15 traps) yielded 18 red squirrels (0.15 captures per trap day) and a single grey squirrel (0.01 captures per trap day). Nine (39%) of the red squirrels caught there in June were present, along with two animals caught and marked previously in adjacent habitats which were now inaccessible. There were seven unchipped squirrels caught: three males and four females. None of the handled animals were reproductively active. Female mean weight was 319 g ± 11.9 s.e. (*n* = 6) and male weight 320 g ± 14.0 s.e. (*n* = 5). A Mann–Whitney U-test did not reveal any significant difference (U = 37, *p* = 0.56), between female red squirrel weights in June and October, when pregnant animals were excluded from the analysis. Male body weight was also not significantly different (U = 30, *p* = 0.68).

On 31 January 2021 another TEM confirmed SQPV case was recorded (APHA Ref 26-M0024-02-21). The 320 g adult male (P6) was caught alive but died whilst being transferred to veterinary quarantine. Erosive skin lesions were present on the chin, lips and around both eyes. There were areas of hair loss on the bridge of the nose, overlying the thoracic inlet and on three digits of the left fore foot and two digits of the right hind foot. It is unclear whether this case was a continuation of autumn 2020 outbreak or was part of a third temporal cluster of infections. In March 2021, only two red squirrels were caught (14 days × 15 traps). These were both females chipped in June 2020 and both had also been caught in October 2020. A single grey squirrel was removed. Later in 2021, an additional male and female marked in June 2020 were recorded along with the recapture of a female red squirrel, first chipped in October 2020. These occurrences meant that at least one of seven (14.2%) October chipped red squirrels and four of the 23 (17.4%) June 2020 marked animals trapped within the limited woodland area accessible (15 traps) in autumn and winter months were known to have been alive there in March 2021.

**Figure 5:**
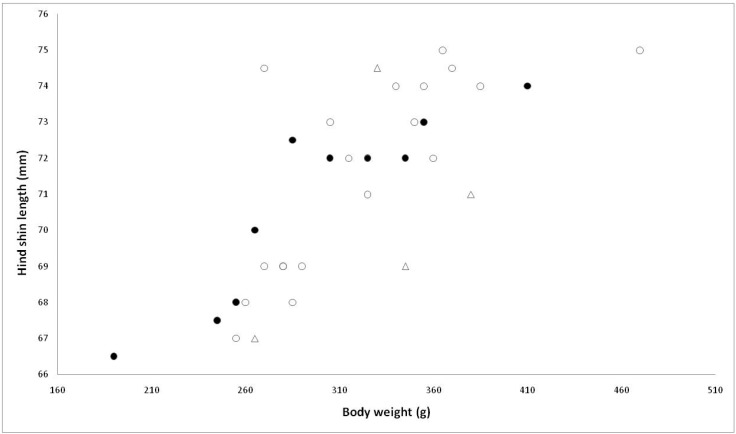
Red squirrel body weight (g) and shin length (mm) during June 2020. Live trapping allowed the partitioning of individual biometrics (weight and shin length) recorded between groups of animals subsequently detected (open triangles) or not found again (open circles). Closed circles—animals trapped in woodland areas that were then inaccessible during October 2020. Open circles—animals trapped in woodland that was later accessible during October 2020 but that were not Scheme 2020. and that were known to be resident there in March 2021 after the autumn/winter squirrelpox outbreak. These encompassed three females and single male.

Live trapping allowed the partitioning of individual biometrics (weight and shin length) recorded between groups of animals subsequently detected (open triangles) or not found again (open circles) (Figure 5). The small detected sample size precludes meaningful statistical comparison with the undetected group although there was a tendency for a larger weight relative to shin length ratio to be observed in detected animals.

## 4. Discussion

Although data collection was opportunistic rather than rigorously experimental, the information collected helped provide valuable insight into the local impact of disease outbreaks on red squirrel population size and individual survival. Despite the limitations of land-access restrictions, mark and recapture data collected before, during and after squirrelpox was first confirmed in 2020 was invaluable. These data provided snapshots of the presence, body weight, size and reproductive state of animals during the outbreak.

The available mark-recapture data strongly suggests a decline in red squirrels associated with squirrelpox infection. It is unclear whether this was associated with a single chronic disease outbreak or successive outbreaks merging temporally. Only 17.4% and 14.2% of animals first marked in June and October 2020, respectively were known to be still alive in March 2021. It should be noted that we were unable to quantify dispersal or completely exclude that individual animals may have remained alive locally but undetected. It is therefore possible that the scale of squirrelpox-associated decline includes animals that remained alive and hence figures represent a maximum rather than absolute mortality. The 17.4% and 14.2% figures equate to 82.6%–85.8% ‘loss’ which is lower than the 93% decrease reported from Tollymore Forest, Northern Ireland between 2008 and 2011 and the estimated reduction to 6% of original density in a Lancashire population [15]. High mortality also reflects Edwards’ [37] observation that only a single female survived a fatal ‘viral’ disease outbreak amongst 30 ‘well-fed’ red squirrels where gross symptoms highly suggested squirrelpox.

Despite confirmation that squirrelpox mortality occurred in the autumn of 2017, autumn of 2020, and the late winter 2020/21, red squirrels persisted on the mainland side of the Menai Strait whilst remaining sympatric with a grey squirrel population suppressed by culling. It is unfortunate that 2017/2018 squirrel trapping data are limited and cannot provide a measure of the scale of red squirrel mortality. In addition, the contribution of ongoing island to mainland dispersal to mainland presence is unclear. Nevertheless, we confirmed that in 2021, four mainland resident females and a male were known to have survived through the previous autumn/late winter outbreak, indicating that mainland population persistence is not solely due to immigration.

Sainsbury et al. [3] report serological evidence from red squirrel carcasses of SQPV exposure, but with concurrent clinical signs absent. Indeed, Chantrey et al. [15] present retrospective serology revealing that 8% of red squirrels exposed to SQPV may survive infection during an epidemic. In the absence of serological studies, it is impossible to know if any of the Gwynedd red squirrels were exposed to SQPV but did not develop disease or alternatively, whether their continued presence is due to simply being lucky and not encountering infection. The small number of animals caught in June 2020 and then later detected precludes meaningful statistical analysis. However, we note that known ‘survivors’ tended to have a relatively high body weight to shin ratio.

Recognising resource limitations in undertaking systematic human observer visual searches in woodland terrain often containing dense fern and ground cover, fallen trees and patches of laurel, the use of a conservation dog was invaluable in carcass detection and recovery. The subsequent successful amplification of SQPV DNA from hair (following, Everest et al. [32]) led to confirmation of an additional squirrelpox case which significantly widened the geographical scale of infection amongst mainland red squirrels in 2020. However, canine detection can only occur in land holdings where access permission is granted. In our scenario, game-bird rearing meant that we could not use a dog to search key woodland areas, and neither could live trapping be undertaken there. This meant that we could also not attempt to remove grey squirrels from the area either, as access permission did not resume until the following February. Our findings are likely to reflect the realities faced in many parts of the UK.

Whether the third confirmed 2020/21 squirrelpox case (found 31 January 2021) derived from a discrete inter-specific infection from grey squirrel to red squirrel or was part of an ongoing intra-specific viral spread (from case 1 on 29 September 2020) is impossible to determine. Carroll et al. [18] document infected red squirrels surviving between 1–22 days whilst Tomkins et al. [11] report survival as 13–17 days from the date of infection, thus we can conclude that the third case was infected in 2021. However, in many respects it is the discovery of a pattern of pathogenic red squirrel cases associated with the loss of the bulk of resident animals that is the headline finding, irrespective of the relative importance of inter and intra-specific viral spread routes. Given the fact that the woodland area contains a mosaic of mixed mature woodland, it would appear unlikely that poor tree seed availability causing population decline was of major significance. It is also notable that mean autumn body weights were good with summer weights similar to the 301–315 g reported by Wauters and Dhondt [38].

The continual detection of successful grey squirrel dispersal onto the island remains a concern because although modelling suggests a resulting squirrelpox outbreak would not cause population extinction [39] the potential loss of genetic variation could have long-term impacts upon population resilience and viability. We believe that our findings offer a useful contribution to the understanding of squirrelpox impacts on red squirrels and reinforce the vital importance of relative geographical isolation in preventing infection spreading from the mainland to Anglesey.

## 5. Conclusions

Our findings demonstrate the value in opportunistic study to further understanding of the impacts of pathogenic infections upon native wildlife. They reaffirm the ongoing threat posed by introduced grey squirrels to red squirrel populations and highlight the need for stringent efforts to be made to exclude grey squirrels from habitats occupied by native red squirrels.

## Data Availability

The data are not publicly available but may be requested by contacting: david.everest@apha.gov.uk.
